# Impact of Total Laboratory Automation on Urine Culture Turnaround Time: A Comparative Study Between Manual Workflow and WASPLab™

**DOI:** 10.3390/diagnostics16081235

**Published:** 2026-04-21

**Authors:** Fizza Khalid, Ahmed J. Alzahrani, Hilal Mohammed, Aymen Khalaf Allah Gamma, Mohamed Elhadi Hassan, Christy Poulose, Azza ElSheikh, Khalid Sumaily, Ahmad Ali Alharbi, Najah Fayyad Aldrous, Mohammed Alsaadan, Mohammed Alnamnakani, Osamah T. Khojah

**Affiliations:** 1 Microbiology Department, MDLab Dr. Sulaiman Al Habib Medical Group, Riyadh 13325, Saudi Arabia; 2 College of Medicine, Al-Imam Mohammed Ibn Saud Islamic University, Riyadh 11432, Saudi Arabia; ahmed.j.alzahrani.imam@gmail.com; 3 Laboratory Department, Dr. Sulaiman Al Habib Medical Group, Riyadh 11643, Saudi Arabia; 4 Department of Basic Medical Sciences, College of Medicine, Majmaah University, Riyadh 11952, Saudi Arabia; 5 Department of Pathology, College of Medicine, King Saud University, Riyadh 11472, Saudi Arabia; 6 Laboratory Department, Chief Medical Officer Office, Dr. Sulaiman Al Habib Medical Group, Riyadh 11643, Saudi Arabia

**Keywords:** Total laboratory automation, Turnaround time, Clinical microbiology workflow, Urine culture, WASPLab

## Abstract

**Background:** Turnaround time (TAT) is a key performance indicator in clinical microbiology, particularly for urine cultures, which represent a high-volume workload and directly impact antimicrobial stewardship. **Methods:** This retrospective observational study compared urine culture TAT before (2023, manual workflow) and after (2025, total laboratory automation using WASPLab™) implementation in a high-volume reference laboratory. A total of 16,210 cultures in 2023 and 60,474 in 2025 were included. TAT was defined as the time from laboratory receipt to final report validation. **Results:** Implementation of total laboratory automation significantly reduced median TAT for both negative cultures (from 49.68 to 34.38 h) and positive cultures (from 50.42 to 34.62 h) (*p* < 0.001). In addition, variability in reporting times decreased, indicating improved consistency. Laboratory productivity increased from 2316 to 7559 cultures per full-time equivalent, representing a 3.26-fold improvement. **Conclusions:** Total laboratory automation significantly improved the speed and consistency of urine culture reporting, supporting enhanced laboratory efficiency and facilitating timely clinical decision-making.

## 1. Introduction

Urinary tract infections (UTIs) are among the most common bacterial infections worldwide and represent a leading cause of outpatient and inpatient antibiotic prescriptions, placing a significant diagnostic burden on clinical microbiology laboratories [1,2]. In Saudi Arabia, UTIs are among the most frequently encountered bacterial infections and are associated with increasing antimicrobial resistance [3]. Antimicrobial resistance represents a major global public health threat, with an estimated 4.95 million deaths associated with bacterial Antimicrobial Resistance (AMR) in 2019 alone [4]. In Saudi Arabia, increasing rates of extended-spectrum β-lactamase (ESBL)-producing Enterobacterales have been reported, reflecting broader regional trends in antimicrobial use and healthcare-associated transmission [5,6].

Urine culture remains the microbiological gold standard for the detection and characterization of urinary pathogens. However, it is important to note that culture results alone cannot reliably distinguish between asymptomatic bacteriuria and symptomatic infection, as this differentiation requires clinical correlation [7]. Similarly, interpretation of potential contamination, particularly in the context of low colony counts or polymicrobial growth, may be challenging and must be considered alongside clinical and patient specific factors. Despite its diagnostic reliability, culture-based identification requires sufficient bacterial growth, inherently limiting rapid clinical decision-making compared with molecular or rapid screening approaches [8]. However, the usefulness of this test is frequently limited by the time required for microbial growth and susceptibility testing. As a result, optimizing TAT is critical for effective antibiotic management and better patient outcomes. Timely reporting of urine culture results is essential for antimicrobial stewardship, as delays often prolong empirical antibiotic therapy. Earlier results enable clinicians to optimize or de-escalate treatment, supporting more targeted patient management.

Conventional microbiology workflows are inherently labor-intensive, with manual inoculation and scheduled “batch” plate reading. These processes are prone to human error and significant variability, especially during high volume periods or staffing shortages [9]. Delays in any phase, from accessioning to determining the final MIC (minimum inhibitory concentration), delay the transition from empirical to targeted therapy.

Total laboratory automation (TLA) has emerged as a transformative solution, integrating specimen processing, smart incubation, and digital imaging into a seamless, 24/7 workflow. Contemporary reviews describe total laboratory automation as a paradigm shift in clinical microbiology, integrating robotics, digital imaging, and continuous incubation to enhance reproducibility and reduce process variability [10]. By utilizing robotic inoculation and high-resolution digital plate reading, TLA systems like WASPLab™ aim to standardize the analytical phase and reduce the “hands-on” time required by technologists [9,11].

Studies have shown that implementation of TLA for urine culture can significantly reduce turnaround times, particularly for preliminary negative results and final reports. For example, a multicohort clinical study found that modifying digital imaging schedules within a TLA system decreased the median time to final result for positive urine cultures from ~71.6 h to ~61.0 h, with similar benefits observed across Gram-negative organisms such as *Escherichia coli* [12]. Another evaluation comparing workflow before and after implementation of a commercial TLA system reported reductions in time to final report, particularly for negative urine cultures, although increased time from receipt to inoculation was observed in certain workflow steps [13,14].

Despite these advantages, TLA adoption is not universally straightforward. Workflow design, staffing patterns, instrument configuration, and laboratory information system integration all influence TLA performance and its impact on TAT. Some studies have reported that automation can increase time for certain processing stages (e.g., receipt to inoculation) if specimens accumulate waiting for automated processing modules, highlighting the importance of workflow optimization [13]. The complexity of these workflow elements should be considered when selecting an appropriate automation system [15]. In addition, optimizing the inoculum size to fit the colony count algorithms for each protocol [16].

Moreover, while TLA can reduce TAT, speed must be balanced with diagnostic accuracy and quality assurance. Shortened incubation and early imaging require validation to ensure that sensitivity and specificity of culture results are not compromised. Studies indicate that earlier digital reading can modestly affect sensitivity at initial time points, emphasizing the need for careful calibration of automated workflows to maintain clinical integrity [12]. By customizing the reading frame defined for imaging as to be optimized for each culture protocol [17].

Given the high volume of urine cultures processed daily and the clinical urgency associated with UTI management, TLA represents a promising strategy to enhance laboratory efficiency and support rapid, reliable diagnostics. However, despite the growing body of evidence demonstrating TLA’s benefits, standardized, multicenter evaluations quantifying its impact specifically on urine culture TAT remain limited. This study seeks to quantify the impact of WASPLab™ implementation on urine culture TAT within a large-scale reference laboratory network. By comparing a full year of manual processing (2023) with a full year of automated processing (2025), we aim to provide a definitive assessment of TLA’s role in modernizing diagnostic microbiology. Despite growing evidence supporting TLA, real-world, large-scale evaluations within high-volume laboratory networks remain limited.

## 2. Methodology

### 2.1. Study Design and Setting

This retrospective, observational study was conducted at the Department of Microbiology, MDLab Dr Sulaiman Al Habib Medical Group. MDLab serves as a centralized reference facility for a large tertiary healthcare network. In 2023, the laboratory supported a 250-bed facility; however, following a network expansion in 2024, the laboratory now supports over 1000 beds across five hospitals and four polyclinics. During the pre-automation period, the laboratory functioned as a moderate-volume microbiology service. By the post-automation period, the laboratory had transitioned into a high-volume, multi-hospital reference laboratory, reflecting a substantial increase in specimen throughput.

Because the laboratory network expanded during the study period, specimen volume increased substantially. To account for this difference, productivity was normalized using the number of cultures processed per full-time equivalent (FTE).

### 2.2. Study Period

Urine culture data were collected during two distinct periods:

- Pre-automation period: January–December 2023 (conventional workflow)
- Post-automation period: January–December 2025 (WASPLab™ workflow)

The year 2024 was excluded from the analysis to account for system installation, software integration, and staff proficiency training.

### 2.3. Specimen Inclusion and Exclusion

All midstream urine and suprapubic aspirates submitted for routine bacterial culture were eligible. Inclusion required a complete electronic timestamp from receipt to final validation. Exclusion criteria included:

- Duplicate specimens from the same patient within a 48-h window.
- Samples rejected for pre-analytical errors (e.g., leakage, insufficient volume).

### 2.4. Workflow Protocols

- Manual Workflow (2023): Specimens were manually accessioned and streaked using calibrated loops onto standard media (Blood Agar, CLED). Plates were incubated in traditional aerobic incubators and manually examined at 18–24 h.
- Automated Workflow (2025): Specimens were processed via the WASPLab™ system. This included automated inoculation using the “E-streak” pattern and continuous incubation in an integrated O_2_ environment. Digital images were automatically captured at 12 and 30 h.

Organism identification was performed using MALDI-TOF mass spectrometry in accordance with routine laboratory practice. Antimicrobial susceptibility testing (AST) was conducted using Vitek 2 Compact System (bioMérieux, Marcy I’Etoile, France) and interpreted according to internationally recognized guidelines (CLSI 2023 and 2025 for each respective years). These procedures were consistent across both study periods.

Full-time equivalent (FTE) staffing was calculated based on contracted working hours of microbiology personnel directly involved in urine culture processing, with one FTE defined as 48 working hours per week in accordance with institutional staffing policy. Staffing levels increased modestly from 7.0 FTE in 2023 to 8.0 FTE in 2025. To account for differences in workload between study periods, urine culture volume was normalized to staffing by calculating the number of cultures processed per FTE per year.

### 2.5. Outcome Measures and Statistical Analysis

The primary outcome was TAT, defined as the time (in hours) from the LIS “received” timestamp to the “final report” validation. Laboratory TAT was calculated from the time of specimen receipt to final result verification. “Received” was defined as the laboratory information system (LIS) accessioning timestamp, representing the moment the specimen physically entered the microbiology laboratory and was logged into the LIS. This timestamp precedes any interaction with the TLA system, including WASPLab^®^ processing.

By using the LIS accessioning time rather than the automated inoculation timestamp, the calculated TAT includes pre-analytical delays, such as specimen queuing prior to automated plating. This approach avoids artificial shortening of reported TAT and is consistent with definitions used in prior laboratory automation studies.

Results were categorized as “Positive” (significant growth requiring ID/AST) or “Negative” (no growth or non-significant growth). Statistical analysis was performed using IBM SPSS Statistics version 25 (IBM Corp., Armonk, NY, USA). Descriptive statistics, including mean, median, standard deviation, and interquartile range (IQR), were calculated to summarize turnaround time data. Given the non-normal distribution of TAT values, comparisons between pre- and post-automation periods were performed using the non-parametric Wilcoxon rank-sum test. A *p*-value of <0.05 was considered statistically significant.

### 2.6. Sample Size

During the study period, a total of:

- 3122 positive urine cultures in 2023 and 11,610 in 2025
- 13,088 negative urine cultures in 2023 and 48,864 in 2025

were included in the final analysis.

### 2.7. Ethical Considerations

This study analyzed anonymized laboratory workflow data collected during routine diagnostic testing. No patient-identifiable information was accessed. In line with institutional policy, informed consent was waived for this retrospective analysis. Ethical approval was obtained from the institutional review board (IRB RC25.12.123).

## 3. Results

A total of urine culture specimens processed during the pre-automation period (2023) and post-automation period (2025) were included in the analysis. Specimens were categorized as positive or negative urine cultures based on routine diagnostic criteria. TAT was defined as the interval from specimen receipt in the laboratory to final result release and was analyzed separately for positive and negative cultures.

**Figure 1:**
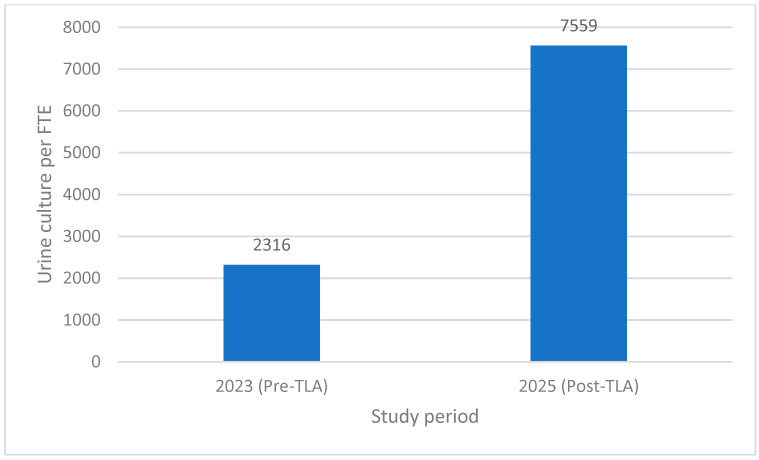
Productivity normalized to staffing (urine culture per FTE).TLA: total laboratory automation; FTE: full-time equivalent.

Total urine culture volume increased from 16,210 specimens in 2023 to 60,474 specimens in 2025. During the same period, staffing increased from 7.0 to 8.0 FTE. When normalized to staffing levels, the number of urine cultures processed per FTE increased from 2316 in 2023 to 7559 in 2025, representing a 3.26-fold increase in productivity following implementation of total laboratory automation as shown in Figure 1.

Productivity is expressed as the number of urine cultures processed per full-time equivalent (FTE) per year. One FTE was defined as 48 working hours per week. Throughput increased from 2316 cultures per FTE in 2023 to 7559 in 2025, representing a 3.26-fold increase following implementation of total laboratory automation.

Following implementation of TLA using the WASPLab™ system, a significant reduction in turnaround time was observed for urine cultures overall. For negative urine cultures, the median TAT in the post-automation period was markedly shorter compared with the pre-automation period, and this reduction was statistically significant (*p* < 0.001). In addition to the reduction in median TAT, the distribution of reporting times for negative cultures was narrower after automation, indicating improved consistency and reduced variability in result reporting. Table 1 summarizes urine culture result distribution and TAT metrics before (2023) and after (2025) implementation of TLA, with proportions calculated within each study year. TAT is calculated from specimen accessioning to final result verification.

Positive urine cultures also demonstrated a statistically significant reduction in turnaround time following TLA implementation (*p* < 0.001). The reduction in TAT for positive cultures was less pronounced than that observed for negative cultures, likely reflecting the additional time required for organism identification and antimicrobial susceptibility testing. The post-automation period showed fewer prolonged delays and a more uniform reporting pattern, reflecting improved workflow standardization. These findings suggest that while downstream processes such as organism identification and antimicrobial susceptibility testing impose inherent time requirements, automation effectively optimized earlier workflow stages, including specimen inoculation, incubation, and initial culture reading.

Comparative analysis of positive and negative urine cultures demonstrated that TLA had a greater impact on negative culture turnaround time, which represents the largest proportion of routine urine specimens. The ability to perform continuous incubation and scheduled digital imaging allowed earlier identification and reporting of no growth results, reducing reliance on fixed manual reading schedules. In contrast, positive cultures benefited primarily from reductions in pre-analytical and analytical delays rather than elimination of required diagnostic steps.

**Figure 2:**
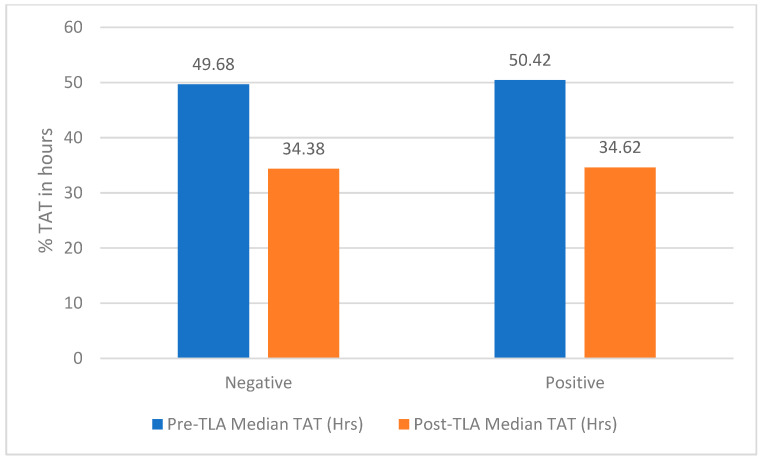
Pre- and Post-TLA Median TAT by Outcome for Routine Urine Cultures. TLA: total laboratory automation; TAT: Turnaround time; Hrs: Hours represent the time of receiving sample and release of the final result.

**Figure 3:**
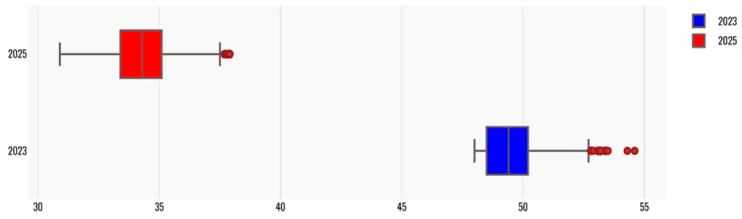
Boxplot distribution of turnaround time for routine urine cultures before and after implementation of total laboratory automation.

Overall, the implementation of TLA was associated with a statistically and operationally significant improvement in urine culture turnaround time as shown in Figure 2 and Figure 3. The observed reductions in TAT, together with decreased variability in reporting times, indicate enhanced laboratory efficiency and workflow reliability in the post-automation period. Given the large sample size, interpretation of study findings focused on absolute changes in turnaround time rather than statistical significance alone. Post-automation implementation was associated with meaningful reductions in median TAT for both negative and positive urine cultures, reflecting improved laboratory efficiency.

TAT is expressed in hours from specimen receipt to final culture result. Boxplots show the median, interquartile range, and overall spread of TAT values for each study year. Statistical comparison between years was performed using the Wilcoxon rank-sum test.

## 4. Discussion

In this study, we observed that in a large, high-volume laboratory, the implementation of TLA with the WASPLab™ system was associated with a substantial reduction in urine culture TAT. The median TAT declined from 50 h in the pre-automation period (2023) to 34 h in the post-automation period (2025), representing an absolute reduction of 18 h (*p* < 0.001).

When stratified by result, both positive and negative cultures benefited from automation: median TAT for positive cultures decreased from 55 h to 43 h, and for negative cultures from 51 h to 34 h (both *p* < 0.001). The magnitude of improvement was larger for negative cultures, consistent with automation driven acceleration of no-growth decision pathways. The large gain in overall TAT occurred alongside substantial growth in the laboratory network’s bed capacity and volume, suggesting that automation can enhance throughput without sacrificing speed. Notably, the proportion of positive urine cultures remained stable across both study periods (approximately 19%), despite a substantial increase in specimen volume. This consistency suggests that the observed improvements in turnaround time were not influenced by shifts in sample size, but rather reflect true workflow efficiency gains following TLA.

These findings are consistent with previous studies evaluating automated microbiology workflows, which consistently report earlier plate reading, improved workflow continuity, and enhanced reporting efficiency [13,14,18,19].

Importantly, the observed reductions in turnaround time occurred despite only a modest increase in staffing. While laboratory service volume increased nearly fourfold, staffing increased by only 1.0 FTE. The more than threefold increase in urine cultures processed per FTE suggests that improvements in laboratory efficiency and turnaround time were likely attributable to total laboratory automation rather than proportional increases in manpower.

The reduction in TAT was most pronounced for negative urine cultures, which constitute the majority of routine specimens. This finding reflects the ability of total laboratory automation to replace batch-based workflows with continuous incubation and scheduled digital imaging, enabling earlier recognition and reporting of no-growth results. Similar observations have been reported in previous studies, where automation primarily reduced non–value-added waiting time rather than accelerating microbial growth [12]. In addition to shortening reporting time, the post-automation period demonstrated reduced variability in TAT, indicating improved workflow consistency and predictability. These improvements are attributable to standardized specimen processing and minimized manual intervention, which together enhance operational efficiency in high-volume laboratory settings [20,21,22].

Our findings are consistent with previous studies demonstrating that total laboratory automation improves turnaround time and workflow efficiency in clinical microbiology laboratories [8,13,17]. For example, Yarbrough et al. reported reductions in time to final report following automation, although they observed potential delays in pre-analytical processing due to specimen queuing [13]. In contrast, such delays were not evident in our setting, likely reflecting differences in workflow configuration, including continuous specimen loading and optimized processing pathways. Similarly, Bailey et al. demonstrated that earlier digital imaging within automated systems contributes to faster reporting, particularly for negative urine cultures [12]. These comparisons suggest that while the overall benefits of automation are consistent, the magnitude of improvement is influenced by local implementation strategies and laboratory workflow design. Notably, the absence of pre-analytical delays in our study contrasts with earlier reports, emphasizing the importance of workflow optimization in maximizing the benefits of automation.

Pre-analytical delays, including specimen receipt-to-inoculation intervals, were not excluded from the analysis. Although such delays have been identified as potential bottlenecks in automated microbiology workflows, their inclusion provides a more realistic assessment of end-to-end laboratory performance under routine operational conditions.

Positive urine cultures also demonstrated a significant, albeit smaller, reduction in TAT after TLA implementation. This finding is consistent with the inherent diagnostic requirements of positive cultures, which include organism identification and antimicrobial susceptibility testing (AST). While these downstream processes impose unavoidable time constraints, TLA optimizes pre-analytical and early analytical stages by standardizing inoculation, minimizing delays to incubation, and ensuring timely first plate review. Previous studies have similarly reported modest but consistent improvements in positive culture TAT following automation, largely attributable to improved workflow standardization and reduced variability in plate handling and reading times [13,23].

Our findings align with prior reports highlighting the benefits of automation for negative urine culture reporting, particularly the reduction in unnecessary incubation time for cultures with no growth [12]. In contrast to earlier studies describing pre-analytical bottlenecks associated with batch processing [13], the implementation of continuous specimen loading and standardized plate handling in our laboratory mitigated these delays and supported sustained TAT improvements at scale.

Importantly, the reduced variability in TAT observed in the post-automation period highlights a key advantage of TLA beyond absolute speed. Variability in reporting times can undermine clinician confidence and complicate antimicrobial decision-making. Reduction in inter-batch variability and improved reporting predictability are recognized quality management advantages associated with automated microbiology platforms [24]. By standardizing processes and reducing dependence on fixed manual workflows, TLA improves the predictability and consistency of urine culture reporting. This effect has been described as a major operational benefit of automated microbiology systems, particularly in high-throughput laboratories [25].

Despite the overall reduction in turnaround time, a subset of cases with prolonged TAT persisted in the post-automation period, as reflected by the long tail in the distribution. These delayed cases likely represent complex clinical or microbiological scenarios rather than workflow inefficiencies alone. Potential contributing factors include polymicrobial growth, low colony counts requiring repeat evaluation, slow-growing or fastidious organisms, and the need for extended incubation or additional identification and antimicrobial susceptibility testing. Pre-analytical factors, such as delayed specimen transport or processing, may also contribute. These findings highlight that while total laboratory automation reduces routine processing time and variability, it does not eliminate delays associated with inherently complex diagnostic cases.

While statistical significance was observed, the clinical relevance of TAT reduction is better reflected by its magnitude and predictability. Earlier reporting of negative urine cultures can substantially reduce unnecessary empiric antibiotic exposure, supporting antimicrobial stewardship efforts. For positive cultures, even modest reductions in TAT improve the consistency of result availability, facilitating earlier antimicrobial optimization and clinical decision-making. Delayed identification of resistant pathogens may lead to inappropriate empiric therapy, which has been associated with worse clinical outcomes and prolonged hospitalization [26].

The observed increase in specimen volume reflects real-world laboratory scale-up rather than differences between equivalent volume environments. Importantly, the sustained improvement in TAT despite increased workload demonstrates the scalability and robustness of total laboratory automation under high-volume operational conditions.

From an operational perspective, TLA enhances workflow standardization and was associated with reduced variability in TAT, as reflected by the narrower distribution and lower dispersion of TAT values observed in the post-automation period. These improvements closely align with antimicrobial stewardship objectives by facilitating earlier microbiological results and supporting evidence-based clinical decision-making [27,28]. While this study did not directly measure operator-dependent factors, the observed reduction in turnaround time variability likely reflects increased process standardization and automation of key workflow steps.

Although this study did not stratify TAT by organism type or Gram classification, differences in microbial growth characteristics inherently influence reporting timelines. Gram-negative organisms, particularly Enterobacterales such as *Escherichia coli*, generally grow more rapidly and are detected earlier than many Gram-positive organisms, which may require longer incubation for definitive identification. TLA standardizes processing through consistent inoculation, continuous incubation, and scheduled digital imaging, enabling earlier and more reliable detection of growth while not altering intrinsic microbial kinetics. Future studies incorporating organism-specific analyses are warranted to further elucidate these effects.

Despite these advantages, the results also underscore that TLA does not eliminate the biological and interpretive constraints inherent to positive urine cultures. AST still requires sufficient bacterial growth and validated interpretation, and automation should not be pursued at the expense of diagnostic accuracy. The observed improvements therefore represent optimization of workflow efficiency rather than artificial acceleration of microbiological processes, supporting the safety and reliability of TLA-based urine culture diagnostics. Beyond workflow acceleration, laboratory automation has been associated with improved cost-efficiency, reduced manual errors, and enhanced standardization of microbiological processes [29].

The strengths of this study include the large sample size and the use of real-world laboratory data following full-scale implementation of Total Laboratory Automation in a high-volume clinical setting. The comparative pre- and post-automation design provides practical insight into the operational impact of automation on laboratory performance.

Rapid molecular diagnostic methods, including PCR-based assays, have significantly reduced turnaround times for pathogen detection, with results often available within a few hours. However, these approaches have important limitations in the context of urinary tract infections. Molecular assays may detect non-viable organisms or colonizing flora and typically provide limited information on antimicrobial susceptibility, particularly for emerging or uncommon resistance mechanisms. In contrast, culture-based diagnostics remain essential for comprehensive organism identification, quantitative assessment of bacterial growth, and phenotypic antimicrobial susceptibility testing. Furthermore, culture allows detection of unexpected or polymicrobial infections that may not be included in targeted molecular panels. Therefore, while molecular methods offer speed, culture remains indispensable for guiding definitive antimicrobial therapy. In this context, TLA does not compete with molecular diagnostics but rather enhances the efficiency, standardization, and clinical utility of culture-based workflows, narrowing the TAT while preserving diagnostic completeness.

Reduced TAT has important implications for antimicrobial stewardship and patient care, enabling earlier optimization and de-escalation of empirical therapy. Improved consistency in reporting further supports timely clinical decision-making and may reduce unnecessary antimicrobial exposure. Although clinical outcomes were not directly assessed, these improvements are likely to positively influence antimicrobial prescribing practices.

Several limitations should be acknowledged. The retrospective, single-center design may limit generalizability to other laboratory settings. In addition, clinical outcome measures such as length of hospital stay or changes in antimicrobial therapy were not evaluated. Future multicenter studies incorporating clinical and economic outcomes would provide further insight into the broader impact of Total Laboratory Automation [30]. The expansion of the hospital network during the study period represents a potential confounding factor, as increased specimen volume may influence workflow dynamics. However, normalization of productivity to FTE and analysis of turnaround time independent of volume were performed to ensure that observed improvements reflect workflow efficiency rather than changes in workload alone. Additionally, exclusion of the implementation year minimized bias related to system validation and staff adaptation. Detailed analysis of day versus night staffing distribution was not performed and represents a limitation. However, unlike conventional batch-based workflows that depend on daytime staffing, total laboratory automation enables continuous specimen processing, incubation, and digital imaging independent of shift patterns, which likely contributes to improved turnaround time and consistency, particularly in high-volume settings.

Finally, while this study evaluates total laboratory automation using the WASPLab™ system, it does not incorporate newer artificial intelligence–driven tools such as PhenoMATRIX^®^, which enable automated interpretation of culture plates and may further enhance diagnostic efficiency. Future studies integrating such technologies and evaluating their impact on both laboratory performance and clinical outcomes would provide a more comprehensive assessment of next-generation microbiology automation. These considerations highlight the need for prospective, multicenter studies incorporating both workflow and clinical endpoints.

Overall, this study demonstrates that total laboratory automation significantly improves urine culture turnaround time while maintaining performance under increasing workload. By capturing pre-analytical delays, emphasizing clinically meaningful effect sizes, and benchmarking findings against existing literature, the results provide a transparent and realistic assessment of TLA impact in a high-volume, real-world microbiology laboratory setting.

## Figures and Tables

**Table 1:** Urine culture outcomes and turnaround time before (2023) and after (2025) implementation of TLA.

Culture Result	Year	Samples (n)	Proportion (%)	Mean TAT (Hours)	Median TAT (Hours)	Min–Max TAT (Hours)	IQ Range	Standard Deviation
Positive	2023	3122	19.26	55.06	50.42	48.0–124.5	10.22	9.34
Positive	2025	11,610	19.20	43.88	34.62	30.13–120.47	3.63	21.14
Negative	2023	13,088	80.74	51.13	49.68	48.0–119.97	2.37	4.31
Negative	2025	48,864	80.80	34.58	34.38	30.13–38.80	1.85	1.68

Percentages are calculated within each study year. Negative cultures include samples reported as no growth, mixed flora, or non-significant growth. TAT values are expressed in hours. IQ: interquartile range; TLA: total laboratory automation.

## Data Availability

The original contributions presented in this study are included in the article. Further inquiries can be directed to the corresponding author.
